# Real-Space Visualization of Canalized Ray Polaritons in a Single Van der Waals Thin Slab

**DOI:** 10.1021/acs.nanolett.4c05277

**Published:** 2025-01-13

**Authors:** Enrique Terán-García, Christian Lanza, Kirill Voronin, Javier Martín-Sánchez, Alexey Y. Nikitin, Aitana Tarazaga Martín-Luengo, Pablo Alonso-González

**Affiliations:** 1 Department of Physics, University of Oviedo, Oviedo 33006, Spain; 2 Center of Research on Nanomaterials and Nanotechnology, CINN (CSIC-Universidad de Oviedo), El Entrego 33940, Spain; 3 Donostia International Physics Center (DIPC), Donostia/San Sebastián 20018, Spain; 4 IKERBASQUEBasque Foundation for Science, Bilbao 48013, Spain

**Keywords:** α-MoO_3_, phonon polaritons, canalization, s-SNOM

## Abstract

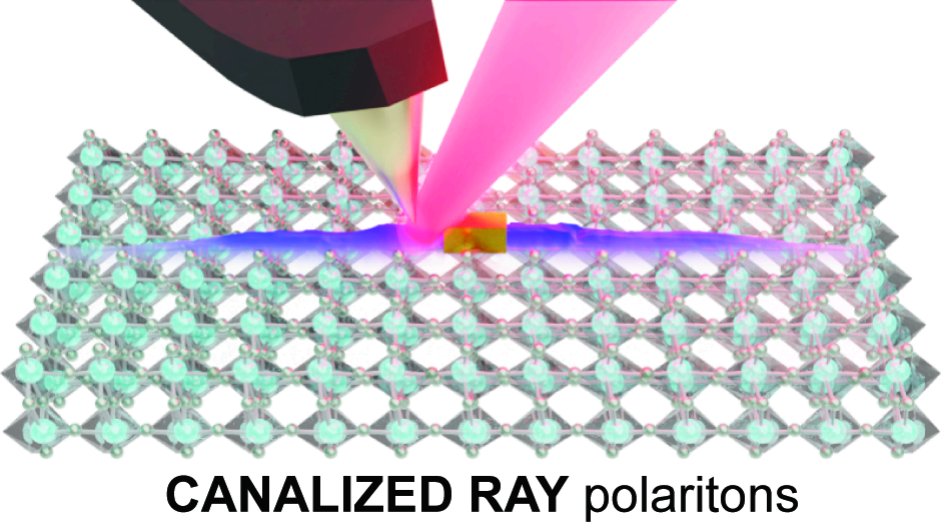

Polaritons are central to the development of nanophotonics, as they provide mechanisms for manipulating light at the nanoscale. A key advancement has been the demonstration of polariton canalization in which the energy flow is directed along a single direction. An intriguing case is the canalization of ray polaritons, characterized by an enhanced density of optical states. Experimental demonstrations of ray polaritons are scarce and their observation in single crystal slabs remains elusive. Here, we propose a novel polaritonic platform based on single thin slabs allowing for the excitation of canalized ray polaritons. By performing near-field nanoimaging, we demonstrate that the necessary conditions for their observation are fulfilled for phonon-polaritons at mid-IR frequencies in thin α-MoO_3_ slabs on SiO_2_ substrates. Our real-space images reveal the propagation of unidirectional phonon-polaritons exhibiting a constant propagating phase. These results might impact the development of compact, low-loss optical nanodevices for applications requiring strong light directionality.

The precise control of light at the nanoscale constitutes one of the main goals in the field of nanophotonics. Recently, the emergence of a large variety of surface polaritons in van der Waals (vdW) crystals^1−3^ has dramatically impacted the field, with demonstrations of strongly directional propagation at the nanoscale. Specifically, phonon polaritons (PhPs)—photons coupled to lattice vibrations—in biaxial vdW crystals, such as α-MoO_3_^4,5^ or α-V_2_O_5_,^6^ are of special interest due to their in-plane hyperbolic propagation, which arises from the different sign of the dielectric permittivity along the two in-plane crystalline directions. Furthermore, when stacking and twisting two^7−10^ or three^11^ slabs of these vdW materials, an exotic type of polaritonic regime arises, characterized by the collimation of the energy flux along a single in-plane direction—the phenomenon known as canalization.^12−16^ As recently reported, polariton canalization can also be achieved through substrate engineering in α-MoO_3_/SiC heterostructures.^17^ Another intriguing type of canalization is the unidirectional propagation of ray polaritons, characterized by a high density of optical states and a constant propagating phase, which has been recently observed in asymmetric twisted vdW stacks.^18^ However, despite the great promises that these phenomena hold for nanophotonics, the complex processes required for the fabrication of twisted stacks hinder their integration in on-chip nanophotonic devices. Therefore, developing a material platform based on pristine single thin slabs that support polariton canalization would be a great step forward, both from a fundamental and applied point of view. Recent theoretical studies based on metamaterials have proposed the phenomenon of loss-induced canalization, which might occur in single slabs given that the canalization direction satisfies the condition |ε| → ∞. Counterintuitively, this latter condition implies that canalization might also occur along crystalline directions with very high optical losses (note the fundamental difference between this case and studies where the direction of high losses is orthogonal to that of the polariton propagation^19^). Although theoretically predicted,^20^ the experimental observation of this type of polariton canalization in single slab platforms remains elusive, even more so for the case of ray polaritons.

Here, we propose and demonstrate loss-induced canalization of ray polaritons in a single slab platform constituted by an in-plane anisotropic thin slab. Specifically, we observe ray PhPs in an α-MoO_3_ thin slab placed on SiO_2_, which propagate with a constant phase along the in-plane [100] direction, as revealed by near-field optical microscopy (s-SNOM). Apart from their fundamental importance, these findings, corroborated by numerical simulations, pave the way for compact applications based on in-plane, constant-phase, ray-like propagation, such as highly directional strong coupling with molecules or integrated nanophotonic circuits.

We first introduce theoretically the phenomenon of loss-induced canalization of ray polaritons in a generic single thin slab exhibiting in-plane anisotropy. To do this, we perform a numerical study for polaritons in a single slab with the dielectric permittivity tensor given by ε = diag(ε_*x*_,ε_*y*_,ε_*z*_). We vary the value of ε_*x*_ from ε_*x*_ = −2 + 0.1*i* to ε_*x*_ = 200*i*, while fixing ε_*y*_ and ε_*z*_. By doing so, we can examine how approaching the |ε| → ∞ condition (in this case along the *x* direction) affects the polariton propagation, potentially inducing the phenomenon of loss-induced canalization.

Specifically, we compute the isofrequency curve (IFC), a slice of the frequency-momentum space defined by a plane of constant frequency,^21^ and the out-of-plane electric field distribution of polaritons in the real space (see “Methods”) using a hypothetical polaritonic material (with a thickness *d* = 200 nm) embedded between two isotropic media (air and substrate). The results obtained show an evolution of the polariton propagation from hyperbolic-like along the *y* direction to highly collimated along the *x* direction while increasing the modulus of ε_*x*_. For ε_*x*_ = 2 + 0.1*i* (Figure 1a), we observe a typical hyperbolic IFC (top panel), denoting a polaritonic propagation restricted to an angular sector centered along the *y*-direction. The white arrows in the figure depict the wavevector **q** defining the direction of the phase propagation and the energy flux **S**—which is orthogonal to the IFC for a fixed **q**—corresponding to hyperbolic rays^22^ (see Supporting Information, Note S2). This hyperbolic propagation is also evident in real space, as shown in the middle panel, where the real part of the out-of-plane electric field Re(E_*z*_) displays hyperbolic-like wavefronts. Furthermore, by taking a field profile along the *y*-direction (dashed-black line in the middle panel), we observe oscillations related to the propagating phase, as typically displayed by hyperbolic polaritons.^23^ Interestingly, when increasing the real and imaginary parts of ε_*x*_ to ε_*x*_ = −20 + 20*i* (Figure 1b), we observe the hyperbolic IFC (top panel) approaching the *y* direction, thus reducing the angular spread of the propagating polaritons. This effect ultimately leads to a change in the ray propagation direction, with most of the wavevectors of the IFC becoming nearly perpendicular to the energy flux (depicted by white arrows). The corresponding distribution of Re(E_*z*_), together with a field profile along the *y* in-plane direction are shown in the middle and bottom panels, respectively. As previously, electric field oscillations are clearly observed. In contrast, when the imaginary part of ε_*x*_ is greatly increased to ε_*x*_ = 200*i* (Figure 1c), i.e., when the condition |ε| → ∞ is approached, the resulting IFC (top panel) shows a significantly decreased eccentricity, flattening along the *y* direction. Such a flat IFC implies the presence of the optical states localized within a very narrow angular sector (see Figure S3 in the Supporting Information) that are characterized by an orthogonality with respect to their corresponding energy flux directions (white arrows in the figure). As a result, the real-space polariton propagation (middle panel) presents a highly directional collimation along the *x* in-plane direction, exhibiting an oscillatory propagating phase (bottom panel), which, however, occurs within very long distances. This finding indicates that (i) the phenomenon of loss-induced canalization of polaritons can take place in generic unstructured single thin polaritonic slabs with in-plane anisotropy, and (ii) the potential emergence of a transition toward a single-phase polariton propagation.

**Figure 1:**
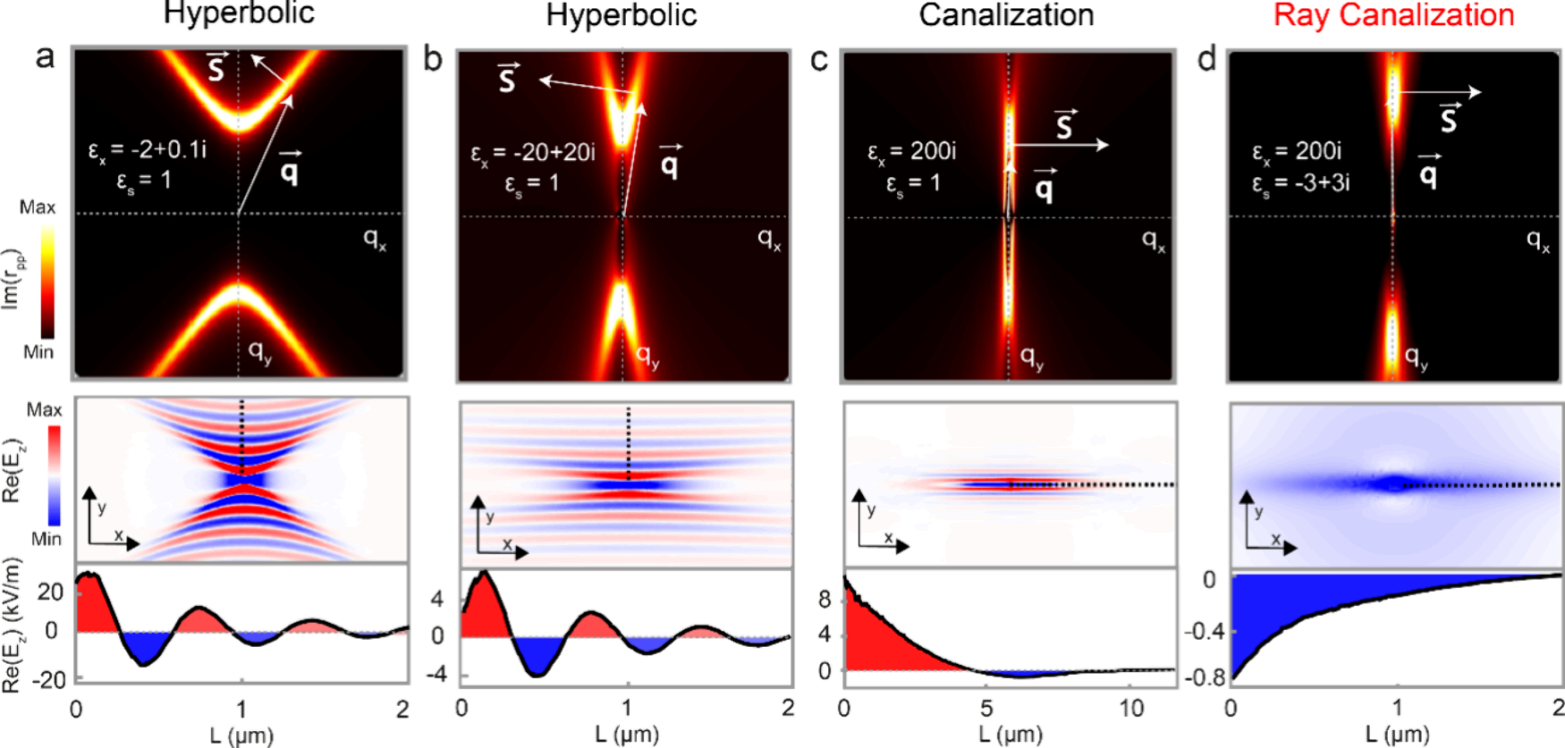
Loss-induced canalization of ray polaritons in a thin in-plane anisotropic slab. (a) Calculated IFC (top panel) and real-space electric field distribution, Re(E_*z*_), (middle panel) of polariton excitations in a single in-plane anisotropic slab with permittivity ε_*x*_ = 2 + 0.1*i*, ε_*y*_ = −1 + 0.1*i*, and ε_*z*_ = 5 + 0.1*i* surrounded by air (ε_*s*_ = 1). A profile along the dashed line in the electric-field distribution showing typical polaritonic oscillations is also included (bottom panel). The thickness of the slab is set to d = 200 nm. (b, c) Same as (a) for ε_*x*_ = −20 + 20*i* and ε_*x*_ = 200*i*, respectively. (d) Same as (c) for a substrate with ε_*s*_ = −3 + 3*i*. The electric field profile shows the propagation of a ray with a fixed phase along the *x* direction.

To further explore this phenomenon, we can also consider the influence of the dielectric environment surrounding the thin slab. To do this, we perform additional calculations keeping the permittivity tensor values of the hypothetical polaritonic material with ε_*x*_ = 200*i* but implementing the presence of a lossy substrate with permittivity ε_*s*_ = −3 + 3*i*. As shown in Figure 1d, this additional consideration has a direct impact on the polaritonic IFC (top panel), which shows now a completely flat line shape giving rise to two important effects: (i) a concentration of the density of allowed optical states along *y* (see Supporting Information, Note S3), and (ii) a complete orthogonality between the wavevectors **q** and their corresponding energy flux vectors **S** (white arrows). The resulting real-space propagation (middle panel) is characterized by a fully collimated polaritonic signal along the *x* in-plane direction displaying a single well-defined propagating phase (bottom panel), i.e., a canalized polaritonic ray. The observation of this exotic propagation in single polaritonic slabs, dependent on both the fulfillment of the |ε_*x*_| → ∞ loss-induced condition and the presence of a moderately metallic substrate (see Supporting Information, Note S4, for further discussion), is still pending.

Interestingly, these conditions can be fulfilled by considering a thin slab of an in-plane anisotropic polar crystal in the vicinity of the Transverse Optical (TO)^1,2^ phonon resonances at mid-infrared frequencies, where Re(ε) → 0 and Im(ε) → ∞, and commonly used substrates in photonics, such as SiO_2_,^24−41^ which exhibits moderate negative permittivity in the same spectral range. To validate this possibility, we consider the polar semiconductor α-MoO_3_, which presents a high structural anisotropy, consisting of bilayers of distorted MoO_6_ octahedra (Figures 2a-b). For each Mo atom, there are three O atoms distinctly linked to it (labeled as O_(1)_, O_(2)_, and O_(3)_ in the figure. See Supporting Information, Note S5, for further discussion). This structural anisotropy gives rise to phonon resonances at different frequency ranges, which exhibit strong dispersion, translating into a large optical anisotropy.^42^ Dipolar modes oriented along the [001] crystal direction and associated with a Mo–O_(2)_ bound stretching^43^ mode (Figure 2a) are related to the appearance of a splitting between the TO phonon resonance at ω = 544 cm^–1^ and the Longitudinal Optical (LO) resonance at ω = 850 cm^–1^ (Figure 2c). This TO-LO splitting leads to the emergence of the so-called Reststrahlen band^44,45^ (RB), in which the permittivity component along the [001] direction reaches negative values (blue region RB1 in Figure 2c), and thus in-plane hyperbolic PhPs can be excited (blue hyperbolic IFC in the inset). At ω_3_ = 821 cm^–1^, another dipolar mode involving a Mo–O_(3)_ bound stretching^43^ mode (Figure 2b) is excited along the [100] direction, whose fingerprint is the TO-LO splitting starting at the TO phonon frequency of 821 cm^–1^. This leads to the formation of another RB,^44,45^ in which the permittivity component along the [100] direction takes negative values (green region RB2 in Figure 2c), and thus in-plane hyperbolic PhPs can also be excited (green hyperbolic IFC in the inset). It is worth noting that from ω_3_ = 821 cm^–1^ (TO phonon frequency along the [100] direction) to ω = 850 cm^–1^ (LO phonon frequency along the [001] direction), both RBs overlap, inducing an elliptical propagation of PhPs (black elliptical IFC in the inset).

**Figure 2:**
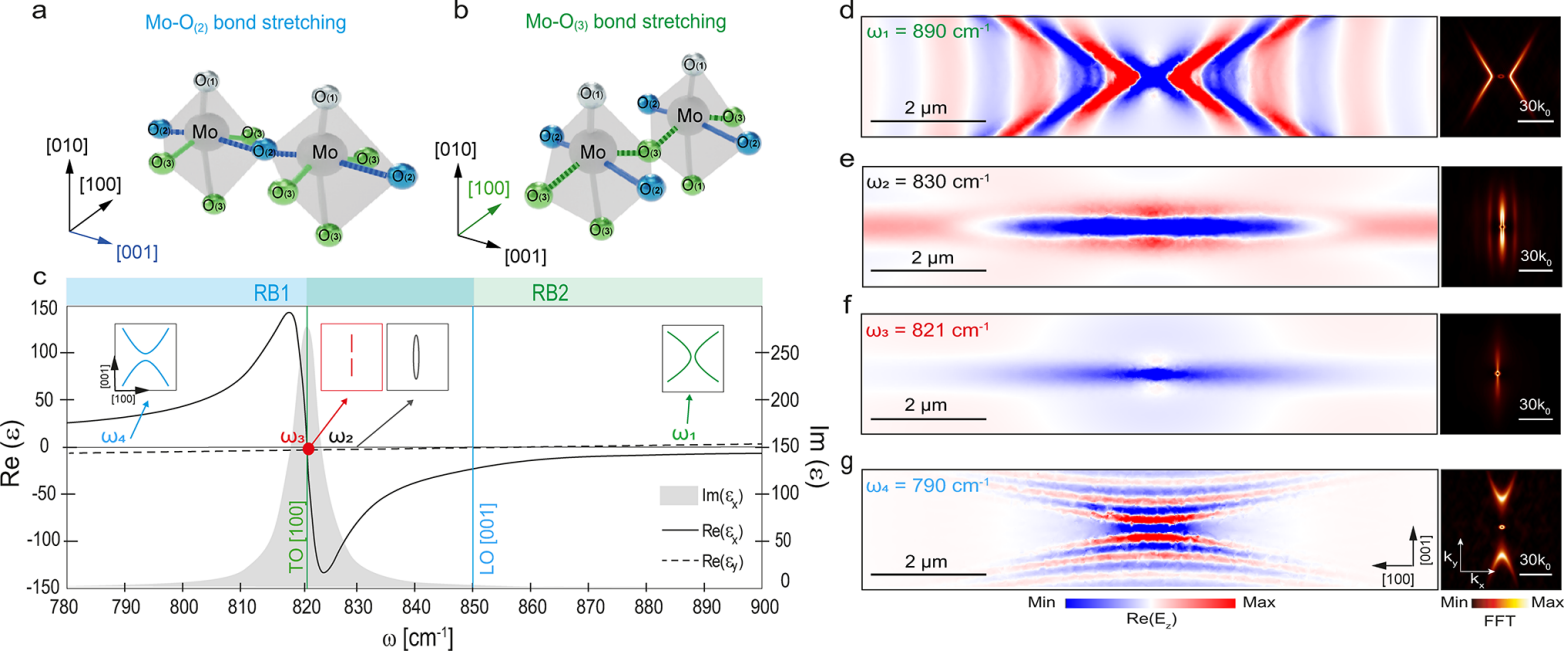
Canalization of ray PhPs in a single thin slab of α-MoO_**3**_. (a-b) Atomic vibrational modes in α-MoO_3_ associated with RB1 (Mo–O_(2)_ bond stretching) and RB2 (Mo–O_(3)_ bond stretching). (c) Real and imaginary part of the α-MoO_3_ dielectric permittivity along the [100] (Re(ε_*x*_), solid line, and Im(ε_*x*_), gray shaded curve, respectively) and real part of the α-MoO_3_ dielectric permittivity along the [001] (Re(ε_*y*_), dashed line) in-plane crystal directions as a function of frequency, ω. The imaginary part of ε along [001] (Im(ε_*y*_), not shown) overlaps with Re(ε_*y*_). The insets represent schematic illustrations of the expected IFCs for each spectral range. (d-g) Numerical simulations of the electric field distribution, Re(E_*z*_), and corresponding Fast Fourier Transform (FFT) (inset) in a 155 nm-thick α-MoO_3_ thin slab (see Supporting Information, Note S6 for a discussion on the slab thickness dependence of the canalized ray PhPs propagation) for ω_1_ = 890 cm^–1^, ω_2_ = 830 cm^–1^, ω_3_ = 821 cm^–1^, and ω_4_ = 790 cm^–1^. The substrate is SiO_2_. A clear canalization of ray PhPs propagating with a fixed phase is observed at ω_3_ = 821 cm^–1^.

Once identified α-MoO_3_ as a suitable in-plane anisotropic material for the observation of canalized ray polaritons, we consider SiO_2_ as a potential substrate, since it exhibits a RB^46^ between 800 and 830 cm^–1^, thus showing a moderately negative permittivity at the α-MoO_3_ TO frequency of ω_3_ = 821 cm^–1^. More precisely, ε_SiO_2__(ω_3_) = −1.3 + 1.2*i*, which fits the necessary condition for obtaining canalized ray polaritons, as shown in Figure 1d. Bearing in mind this α-MoO_3_/SiO_2_ material configuration, we perform numerical simulations to investigate the propagation of PhPs. The results obtained, displayed in Figures 2d–g, show an evolution of the PhP propagation from hyperbolic-like along the [100] in-plane direction to highly collimated along the same direction while decreasing the excitation frequency from ω_1_ = 890 cm^–1^ (Figure 2d) to ω_3_ = 821 cm^–1^ (Figure 2f), i.e., at the RB2 TO phonon frequency. Specifically, as expected at frequencies belonging to the α-MoO_3_ RB2, at ω_1_ we observe hyperbolic wavefronts centered along the [100] in-plane direction. This hyperbolic behavior is further confirmed by the shape of the corresponding IFC (inset in Figure 2d). By decreasing the frequency to the spectral range overlapping between RB2 and RB1, e.g. ω_2_ = 830 cm^–1^, the angular spreading of the hyperbolic wavefronts slowly reduces (Figure 2e), and elliptic-like propagation is observed, which is corroborated by the corresponding IFC (right inset). Intriguingly, by lowering the excitation frequency further to the TO frequency, we observe a markedly different type of propagation characterized by a complete PhP collimation along the [100] in-plane crystal direction with a constant phase (Figure 2f) (see Supporting Information, Note S7 for further discussion). The corresponding IFC (right inset) is an open curve that shows a density of optical states concentrated along the *y* direction. As a result, all allowed wavevectors are completely orthogonal to the energy flux direction, ensuring the constant-phase ray-like nature of the collimated propagation. Finally, decreasing the frequency further, at ω_4_, which lies inside the hyperbolic RB1, we observe a hyperbolic-like real-space PhP propagation (Figure 2g) centered along the [001] in-plane crystal direction, as confirmed by the corresponding IFC (inset in Figure 2g). Collectively, these results validate our prediction (Figure 1d) of a novel type of PhP propagation in thin polar slabs at the TO phonon frequency, driven by the exceptionally large imaginary part of the permittivity component in the propagation direction—*loss-induced canalization*. This phenomenon canalizes the PhP propagation (see Supporting Information, Note S8), while a moderate value of the substrate permittivity enables the constant-phase characteristic, resulting in canalization of ray PhPs. Remarkably, the propagation length, L_p_, of these canalized ray PhPs in α-MoO_3_ reaches values of about 2 μm (see Figure S7 in the Supporting Information), which promises potential uses for sensing applications using nanocavities. Interestingly, this L_p_ is not influenced by the layer thickness, which reflects the intrinsic surface nature of these polaritonic excitations.

To experimentally demonstrate the existence of canalized ray PhPs predicted in Figure 2f, we conducted scattering-type scanning near-field optical microscopy (s-SNOM) to image PhPs (see “Methods”) excited in a single thin slab of α-MoO_3_ (d = 155 nm) on a SiO_2_ substrate (thus replicating the parameters in the numerical simulations of Figure 2d–f). A sketch of the experimental setup and an optical image of the sample employed is shown in Figure 3a (left and right, respectively).

**Figure 3:**
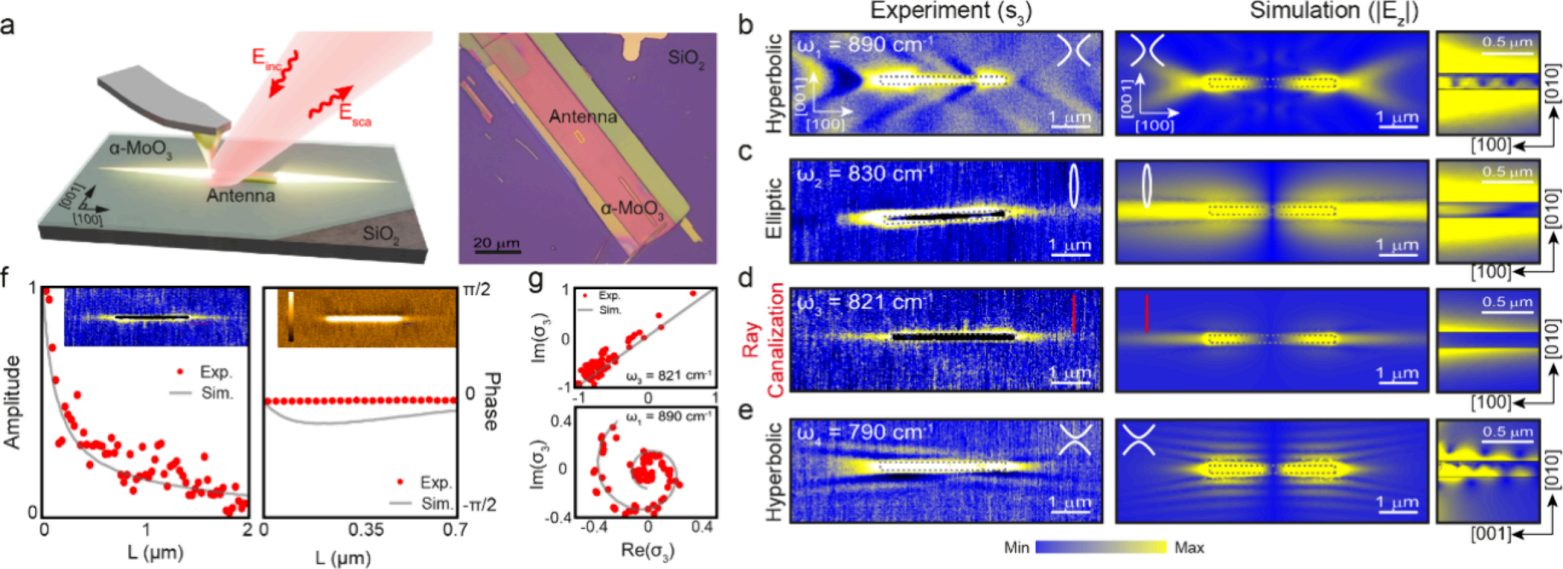
Real-space observation of canalized ray PhPs in a single thin slab of α-MoO_**3**_. (a) Left: Schematics of the s-SNOM near-field visualization of PhPs in a single thin slab of α-MoO_3_ placed on top of a SiO_2_ substrate. *E*_inc_ and *E*_sca_ represent the incident and scattered electromagnetic field in s-SNOM. Right: Optical image of the 155 nm-thick α-MoO_3_ single thin slab (red area) placed on SiO_2_. The gold rod nanoantenna used for an efficient excitation of PhPs is highlighted with a yellow dotted line. (b–e) Left: Near-field amplitude s-SNOM images (s_3_) taken at ω_1_ = 890 cm^–1^, ω_2_ = 830 cm^–1^, ω_3_ = 821 cm^–1^ and ω_4_ = 790 cm^–1^, respectively. Right. Numerically simulated out-of-plane electric field |E_*z*_| obtained by mimicking the experiment. A good agreement between experiment and theory is obtained. The insets to the right represent |E_*z*_| in the *xz* plane in (b–d), and *yz* plane in (e). (f) Left: Profile of the near-field amplitude (s_3_) (red symbols) obtained along the [100] direction at 821 cm^–1^. The solid gray line represents the numerically calculated profile mimicking the experiment. Right. Same than left for the near-field phase. (g) Complex plane representation (real vs imaginary part) of the experimental near**-**field profile, σ_3_ (see “Methods”), at ω_1_ = 890 cm^–1^ (bottom) and ω_3_ = 821 cm^–1^ (top), revealing the oscillatory/nonoscillatory phase propagation of PhPs, respectively. Red dots denote the experimental data, while solid gray lines represent the numerical calculations.

To efficiently launch the PhPs, we fabricate a gold rod nanoantenna on top of the α-MoO_3_ single thin slab with its longitudinal axis aligned along the [100] in-plane direction of the crystal (see “Methods”) and we illuminate it with p-polarized mid-infrared light. This optical scheme enables the s-SNOM tip to function mainly as a near-field probe allowing for a direct visualization of the real-space propagation of polaritons, as previously reported.^47−51^ The left panels of Figure 3b–e display the resulting near-field images at four illuminating frequencies ranging from 890 to 790 cm^–1^. At ω_1_ = 890 cm^–1^ (Figure 3b), we observe hyperbolic wavefronts emanating from the Au nanoantenna along the α-MoO_3_ [100] direction, as expected for PhPs in the RB2, and corroborated by numerical simulations mimicking the experimental conditions (middle panel). By plotting the simulated |E_*z*_| in the *xz*-plane (right panel), we can also clearly observe the volume nature (zigzag pattern) of the excited polaritonic mode at this frequency. A very different PhP propagation is observed when decreasing the illuminating frequency to ω_2_ = 830 cm^–1^ (Figure 3c). In this case, PhPs appear propagating along all directions in the plane, although with a clear anisotropy, indicating an elliptic in-plane propagation. This propagation is corroborated by numerically simulated |E_*z*_| in the *xy*-plane (middle panel), and in the *xz*-plane (right panel), the latter again showing a volume-like character of the polaritonic mode, although now with much longer field oscillations. More interestingly, at ω_3_ = 821 cm^–1^ (Figure 3d), i.e., in the vicinity of the RB2 TO frequency, we observe that the near-field signal is fully collimated along the [100] in-plane direction. Moreover, this signal reveals the PhP propagation through the material with a constant phase, as no oscillations are observed in the near-field signal (Figure 3f, left and right panels for the amplitude and phase, respectively), which is corroborated by numerical simulations (middle panel in Figure 3d). Notably, the simulated |E_*z*_| in the *xz*-plane also shows that the polariton penetration in the material is negligible at this frequency, revealing the transition to an optical mode with a surface-like character, such as expected for an in-plane polaritonic ray.^52^ The unique characteristics of such ray PhPs can be better appreciated by representing line profiles of the complex near-field signal (see “Methods”),^53^ as shown in Figure 3g for the canalization frequency (symbols in the top panel of Figure 3g obtained by extracting a profile along the red-dashed lines in the insets of Figure 3f). The plotted s_3_ at ω_3_ reveals a line arising from the constant phase of the exponentially decaying polaritonic field. In contrast, for the frequency ω_1_ corresponding to hyperbolic propagation, a radically different curve in the form of a counterclockwise rotating spiral is observed (bottom panel), indicative of an oscillating field. Gray solid lines in Figure 3g represent numerical simulations and corroborate our experimental observations. Finally, measuring at a lower illuminating frequency of ω_4_ = 790 cm^–1^ (Figure 3e), a hyperbolic-like PhP propagation is recovered, as can be observed in the corresponding near-field image (left panel), where the hyperbolic wavefronts emanate from the long axis of the Au nanoantenna. This PhP propagation, also evident in the numerical simulations displayed in the middle panel, is expected for PhPs in the α-MoO_3_ RB1. Additionally, the numerically calculated |E_*z*_| in the *yz*-plane plotted in the right panel reveals the characteristic zigzag pattern originated by volumetric PhP hyperbolic modes at this frequency.

Overall, our results provide unambiguous demonstration of canalized ray PhPs in an unstructured single thin slab. These exotic PhPs are driven by the large imaginary part of the α-MoO_3_ permittivity along the propagation direction (loss-induced canalization) and enhanced by a moderate negative permittivity of the substrate (SiO_2_). Our results open new avenues for the design of polaritonic devices that leverage canalized ray polaritons in applications such as thermal transport,^54−57^ integrated nanophotonic circuits^58^ or molecular sensing,^59,60^ as well as for fundamental studies of strong light-matter interactions. Ultimately, our study demonstrates the possibility of achieving advanced and precise nanoscale light control in simple, unstructured material systems on widely used substrates, offering a direct pathway to implement these findings in future technological applications.

## Supporting Information Available

Additional studies including a detailed description of the experimental and numerical methods employed to obtain the main results of the work, the calculation of the polariton electric field distribution and the Poynting vector direction, a quantitative analysis of the density of optical states (DOS), the investigation of the role of the substrate in the canalization of ray PhPs, discussion of the structural anisotropy of α-MoO_3_, the study of the influence of slab thickness on the propagation of canalized ray PhPs in α-MoO_3_/SiO_2_, confirmation of the constant propagating phase of canalized ray polaritons and the investigation of the influence of material losses along the α-MoO_3_ [100] in-plane direction on the propagation of canalized ray PhPs in α-MoO_3_/SiO_2_ (PDF)
Numerical simulations of dipole-launched PhPs in α-MoO_3_/SiO_2_ at 821 cm^−1^ varying the imaginary part of the α-MoO_3_ permittivity along the [100] in-plane direction (AVI)

## Supplementary Material

nl4c05277_si_001.pdf

nl4c05277_si_002.avi

## References

[ref1] Basov D. N.; et al. Polaritons in van der Waals materials. Science 2016, 354, aag1992 10.1126/science.aag1992. 27738142

[ref2] Low T.; Chaves A.; Caldwell J.; et al. Polaritons in layered two-dimensional materials. Nat. Mater. 2017, 16, 182–194. 10.1038/nmat4792. 27893724

[ref3] Guo X.; Lyu W.; Chen T.; Luo Y.; Wu C.; Yang B.; Sun Z.; Garcia de Abajo F. J.; Yang X.; Dai Q. Polaritons in Van der Waals Heterostructures. Adv. Mater. 2023, 35, 2201856 10.1002/adma.202201856. 36121344

[ref4] Ma W.; Alonso-González P.; Li S.; et al. In-plane anisotropic and ultra-low-loss polaritons in a natural van der Waals crystal. Nature 2018, 562, 557–562. 10.1038/s41586-018-0618-9. 30356185

[ref5] Zheng Z.; et al. A mid-infrared biaxial hyperbolic van der Waals crystal. Sci. Adv. 2019, 5, eaav8690 10.1126/sciadv.aav8690. 31139747 PMC6534390

[ref6] Taboada-Gutiérrez J.; Álvarez-Pérez G.; Duan J.; et al. Broad spectral tuning of ultra-low-loss polaritons in a van der Waals crystal by intercalation. Nat. Mater. 2020, 19, 964–968. 10.1038/s41563-020-0665-0. 32284598

[ref7] Hu G.; Ou Q.; Si G.; et al. Topological polaritons and photonic magic angles in twisted α-MoO_3_ bilayers. Nature. 2020, 582, 209–213. 10.1038/s41586-020-2359-9. 32528096

[ref8] Chen M.; Lin X.; Dinh T. H.; et al. Configurable phonon polaritons in twisted α-MoO_3_. Nat. Mater. 2020, 19, 1307–1311. 10.1038/s41563-020-0732-6. 32661384

[ref9] Zheng Z.; et al. Phonon Polaritons in Twisted Double-Layers of Hyperbolic van der Waals. Nano Lett. 2020, 20 (7), 5301–5308. 10.1021/acs.nanolett.0c01627. 32574060

[ref10] Duan J.; et al. Twisted nano-optics: manipulating light at the nanoscale with twisted phonon polaritonic slabs. Nano Lett. 2020, 20 (7), 5323–5329. 10.1021/acs.nanolett.0c01673. 32530634

[ref11] Duan J.; Álvarez-Pérez G.; Lanza C.; et al. Multiple and spectrally robust photonic magic angles in reconfigurable α-MoO_3_ trilayers. Nat. Mater. 2023, 22, 867–872. 10.1038/s41563-023-01582-5. 37349399

[ref12] Gomez-Diaz J. S.; Alù A. Flatland Optics with Hyperbolic Metasurfaces. ACS Photonics 2016, 3 (12), 2211–2224. 10.1021/acsphotonics.6b00645.

[ref13] Gomez-Diaz J. S.; Tymchenko M.; Alù A. Hyperbolic Plasmons and Topological Transitions Over Uniaxial Metasurfaces. Phys. Rev. Lett. 2015, 114, 233901 10.1103/PhysRevLett.114.233901. 26196803

[ref14] Feng S. Loss-Induced Omnidirectional Bending to the Normal in ϵ-Near-Zero Metamaterials. Phys. Rev. Lett. 2012, 108, 193904 10.1103/PhysRevLett.108.193904. 23003042

[ref15] Chang P. H.; Lin C.; Helmy A. S. Field canalization using anisotropic 2D plasmonics. npj 2D Mater. Appl. 2022, 6, 5 10.1038/s41699-021-00283-4.

[ref16] Li P.; Hu G.; Dolado I.; et al. Collective near-field coupling and nonlocal phenomena in infrared-phononic metasurfaces for nano-light canalization. Nat. Commun. 2020, 11, 3663 10.1038/s41467-020-17425-9. 32694591 PMC7374561

[ref17] Duan J.; Tarazaga Martín-Luengo A.; Lanza C.; Partel S.; Voronin K.; Tresguerres-Mata A. I. F.; Álvarez-Pérez G.; Nikitin A. Y.; Martín-Sánchez J.; Alonso-González P. Canalization-based super-resolution imaging using a single van der Waals layer. arXiv 2024, 10.48550/arXiv.2404.14876. PMC13109927 39937910

[ref18] Álvarez-Cuervo J.; Obst M.; Dixit S.; et al. Unidirectional ray polaritons in twisted asymmetric stacks. Nat. Commun. 2024, 15, 9042 10.1038/s41467-024-52750-3. 39426947 PMC11490623

[ref19] F. Tresguerres-Mata A. I.; Lanza C.; Taboada-Gutiérrez J.; et al. Observation of naturally canalized phonon polaritons in LiV_2_O_5_ thin layers. Nat. Commun. 2024, 15, 2696 10.1038/s41467-024-46935-z. 38538588 PMC10973474

[ref20] Correas-Serrano D.; Alù A.; Gomez-Diaz J. S. Plasmon canalization and tunneling over anisotropic metasurfaces. Phys. Rev. B 2017, 96, 075436 10.1103/PhysRevB.96.075436.

[ref21] Álvarez-Pérez G.; et al. Negative reflection of nanoscale-confined polaritons in a low-loss natural medium. Sci. Adv. 2022, 8, eabp8486 10.1126/sciadv.abp8486. 35857836 PMC9299554

[ref22] Martín-Sánchez J.; et al. Focusing of in-plane hyperbolic polaritons in van der Waals crystals with tailored infrared nanoantennas. Sci. Adv. 2021, 7, eabj0127 10.1126/sciadv.abj0127. 34623915 PMC8500510

[ref23] Álvarez-Pérez G.; et al. Active Tuning of Highly Anisotropic Phonon Polaritons in Van der Waals Crystal Slabs by Gated Graphene. ACS Photonics 2022, 9 (2), 383–390. 10.1021/acsphotonics.1c01549.

[ref24] Dai S.; et al. Tunable Phonon Polaritons in Atomically Thin van der Waals Crystals of Boron Nitride. Science 2014, 343, 1125–1129. 10.1126/science.1246833. 24604197

[ref25] Giles A.; Dai S.; Vurgaftman I.; et al. Ultralow-loss polaritons in isotopically pure boron nitride. Nat. Mater. 2018, 17, 134–139. 10.1038/nmat5047. 29251721

[ref26] Caldwell J. D.; Aharonovich I.; Cassabois G.; et al. Photonics with hexagonal boron nitride. Nat. Rev. Mater. 2019, 4, 552–567. 10.1038/s41578-019-0124-1.

[ref27] Sternbach A. J.; et al. Negative refraction in hyperbolic hetero-bicrystals. Science 2023, 379, 555–557. 10.1126/science.adf1065. 36758086

[ref28] Chen J.; Badioli M.; Alonso-González P.; et al. Optical nano-imaging of gate-tunable graphene plasmons. Nature 2012, 487, 77–81. 10.1038/nature11254. 22722861

[ref29] Woessner A.; Lundeberg M.; Gao Y.; et al. Highly confined low-loss plasmons in graphene–boron nitride heterostructures. Nat. Mater. 2015, 14, 421–425. 10.1038/nmat4169. 25532073

[ref30] Nikitin A.; Alonso-González P.; Vélez S.; et al. Real-space mapping of tailored sheet and edge plasmons in graphene nanoresonators. Nature Photon 2016, 10, 239–243. 10.1038/nphoton.2016.44.

[ref31] Bezares F. J.; et al. Intrinsic Plasmon–Phonon Interactions in Highly Doped Graphene: A Near-Field Imaging Study. Nano Lett. 2017, 17 (10), 5908–591. 10.1021/acs.nanolett.7b01603. 28809573

[ref32] Duan J.; Álvarez-Pérez G.; Tresguerres-Mata A. I. F.; et al. Planar refraction and lensing of highly confined polaritons in anisotropic media. Nat. Commun. 2021, 12, 4325 10.1038/s41467-021-24599-3. 34267201 PMC8282686

[ref33] Taboada-Gutiérrez J.; et al. Unveiling the Mechanism of Phonon-Polariton Damping in α-MoO_3_. ACS Photonics 2024, 11 (9), 3570–3577. 10.1021/acsphotonics.4c00485. 39310295 PMC11413844

[ref34] Li P.; et al. Optical Nanoimaging of Hyperbolic Surface Polaritons at the Edges of van der Waals Materials. Nano Lett. 2017, 17 (1), 228–235. 10.1021/acs.nanolett.6b03920. 27966994

[ref35] Vyshnevyy A. A.; et al. van der Waals Materials for Overcoming Fundamental Limitations in Photonic Integrated Circuitry. Nano Lett. 2023, 23 (17), 8057–8064. 10.1021/acs.nanolett.3c02051. 37615652

[ref36] Maia F. C. B.; et al. Anisotropic Flow Control and Gate Modulation of Hybrid Phonon-Polaritons. Nano Lett. 2019, 19 (2), 708–715. 10.1021/acs.nanolett.8b03732. 30668122

[ref37] Feres F. H.; et al. Graphene Nano-Optics in the Terahertz Gap. Nano Lett. 2023, 23 (9), 3913–3920. 10.1021/acs.nanolett.3c00578. 37126430

[ref38] Fali A.; et al. Refractive Index-Based Control of Hyperbolic Phonon-Polariton Propagation. Nano Lett. 2019, 19 (11), 7725–7734. 10.1021/acs.nanolett.9b02651. 31650843

[ref39] Duan J.; et al. Active and Passive Tuning of Ultranarrow Resonances in Polaritonic Nanoantennas. Adv. Mater. 2022, 34, 2104954 10.1002/adma.202104954. 34964174

[ref40] Caldwell J.; Kretinin A.; Chen Y. Sub-diffractional volume-confined polaritons in the natural hyperbolic material hexagonal boron nitride. Nat. Commun. 2014, 5, 5221 10.1038/ncomms6221. 25323633

[ref41] Mayer R. A.; et al. Paratellurite Nanowires as a Versatile Material for THz Phonon Polaritons. ACS Photonics 2024, 11 (10), 4323–4333. 10.1021/acsphotonics.4c01249.

[ref42] Galiffi E.; Carini G.; Ni X.; et al. Extreme light confinement and control in low-symmetry phonon-polaritonic crystals. Nat. Rev. Mater. 2024, 9, 9–28. 10.1038/s41578-023-00620-7.

[ref43] Py M. A.; Schmid P. E.; Vallin J. T. Raman scattering and structural properties of MoO_3_. Nuov Cim B 1977, 38, 271–279. 10.1007/BF02723496.

[ref44] Álvarez-Pérez G.; et al. Infrared Permittivity of the Biaxial van der Waals Semiconductor α-MoO_3_ from Near- and Far-Field Correlative Studies. Adv. Mater. 2020, 32, 1908176 10.1002/adma.201908176. 32495483

[ref45] Sarkar M.; Enders M. T.; Shokooh-Saremi M.; Watanabe K.; Taniguchi T.; Sheinfux H. H.; Koppens F. H. L.; Papadakis G. T. Retrieving optical parameters of emerging van der Waals flakes. arXiv 2023, 10.48550/arXiv.2305.13994.

[ref46] Aguilar-Merino P.; Álvarez-Pérez G.; Taboada-Gutiérrez J.; et al. Extracting the Infrared Permittivity of SiO_2_ Substrates Locally by Near-Field Imaging of Phonon Polaritons in a van der Waals Crystal. Nanomaterials 2021, 11, 120 10.3390/nano11010120. 33430225 PMC7825664

[ref47] Dai S.; et al. Efficiency of Launching Highly Confined Polaritons by Infrared Light Incident on a Hyperbolic Material. Nano Lett. 2017, 17 (9), 5285–5290. 10.1021/acs.nanolett.7b01587. 28805397

[ref48] Alonso-González P.; et al. Controlling graphene plasmons with resonant metal antennas and spatial conductivity patterns. Science 2014, 344, 1369–1373. 10.1126/science.1253202. 24855026

[ref49] Alonso-González P.; Albella P.; Schnell M.; et al. Resolving the electromagnetic mechanism of surface-enhanced light scattering at single hot spots. Nat. Commun. 2012, 3, 684 10.1038/ncomms1674. 22353715 PMC3293409

[ref50] Pons-Valencia P.; Alfaro-Mozaz F. J.; Wiecha M. M.; et al. Launching of hyperbolic phonon-polaritons in h-BN slabs by resonant metal plasmonic antennas. Nat. Commun. 2019, 10, 3242 10.1038/s41467-019-11143-7. 31324759 PMC6642108

[ref51] Duan J.; et al. Enabling propagation of anisotropic polaritons along forbidden directions via a topological transition. Sci. Adv. 2021, 7, eabf2690 10.1126/sciadv.abf2690. 33811076 PMC11060020

[ref52] Voronin K. V.; et al. Fundamentals of Polaritons in Strongly Anisotropic Thin Crystal Layers. ACS Photonics 2024, 11 (2), 550–560. 10.1021/acsphotonics.3c01428.

[ref53] Yoxall E.; Schnell M.; Nikitin A.; et al. Direct observation of ultraslow hyperbolic polariton propagation with negative phase velocity. Nature Photon 2015, 9, 674–678. 10.1038/nphoton.2015.166.

[ref54] Sarkar M.; Haldankar R.; Legendre L.; Davidova G.; Bachtold A.; Papadakis G. T. Coherent Thermal Emission from Large-Scale Suspended Nanomechanical Membranes. arXiv 2024, 10.48550/arXiv.2409.03045.

[ref55] Chistyakov V. A.; et al. Thermal Emission Control via Twist Tuning of Embedded Eigenstates in α-MoO_3_ Nanostructures. ACS Appl. Nano Mater. 2024, 7 (2), 1519–1525. 10.1021/acsanm.3c03076.

[ref56] Sarkar M.; et al. Lithography-free directional control of thermal emission. Nanophotonics. 2024, 13 (5), 763–771. 10.1515/nanoph-2023-0595. 39635103 PMC11502043

[ref57] Yang S.-H.; et al. Anisotropic radiative heat transfer between nanoparticles mediated by a twisted bilayer graphene grating. Phys. Rev. B 2021, 104, 125417 10.1103/PhysRevB.104.125417.

[ref58] Soto Lamata I.; et al. Plasmons in Cylindrical 2D Materials as a Platform for Nanophotonic Circuits. ACS Photonics 2015, 2 (2), 280–286. 10.1021/ph500377u.

[ref59] Bylinkin A.; et al. Dual-Band Coupling of Phonon and Surface Plasmon Polaritons with Vibrational and Electronic Excitations in Molecules. Nano Lett. 2023, 23 (9), 3985–399. 10.1021/acs.nanolett.3c00768. 37116103

[ref60] Bylinkin A.; Castilla S.; Slipchenko T. M.; et al. On-chip phonon-enhanced IR near-field detection of molecular vibrations. Nat. Commun. 2024, 15, 8907 10.1038/s41467-024-53182-9. 39414807 PMC11484778

