# Supplementary material for: Real-Space Visualization of Canalized Ray Polaritons in a Single Van der Waals Thin Slab
Source: Nano Lett. 2025 Jan 13;25(6):2203–9. doi: 10.1021/acs.nanolett.4c05277 (PMC11827108; doi:10.1021/acs.nanolett.4c05277)
Supplement: Supplementary file 1 — nl4c05277_si_001.pdf [file nl4c05277_si_001.pdf]

Supporting information for

Real-space visualization of Canalized Ray Polaritons in a single van der Waals thin slab

Enrique Terán-García[1,2,†], Christian Lanza[1,2,†], Kirill Voronin[3], Javier Martín-Sánchez[1,2], Alexey Y. Nikitin[3,4], Aitana Tarazaga Martín-Luengo[1,2,*] and Pablo Alonso-González[1,2,*]

[1]*Department of Physics, University of Oviedo, Oviedo 33006, Spain.*
[2]*Center of Research on Nanomaterials and Nanotechnology, CINN (CSIC-Universidad de Oviedo), El Entrego 33940, Spain.*
[3]*Donostia International Physics Center (DIPC), Donostia/San Sebastián 20018, Spain.*
[4]*IKERBASQUE, Basque Foundation for Science, Bilbao 48013, Spain.*

[*]aitanatarazaga@uniovi.es, pabloalonso@uniovi.es
[†] *These authors contributed equally to this work.*

The Supporting Information of this work consists of eight sections displaying complementary results to those described in the main manuscript and is structured as follows:

- **Note S1.** Methods.
- **Note S2.** Calculation of the polariton electric field distribution and the Poynting vector direction.
- **Note S3.** Quantitative analysis of the Density of Optical States (DOS).
- **Note S4.** Role of the substrate in the canalization of ray PhPs.
- **Note S5.** Discussion of the structural anisotropy of α-MoO_3.
- **Note S6.** Investigation of the influence of slab thickness on the propagation of canalized ray PhPs in α-MoO_3/SiO_2.
- **Note S7.** Constant propagating phase of canalized ray polaritons.
- **Note S8.** Investigation of the influence of material losses along the α-MoO_3 [100] in-plane direction on the propagation of canalized ray PhPs in α-MoO_3/SiO_2.

Note S1. Methods.

In this Note, we provide a detailed description of the experimental and numerical methods employed to obtain the results discussed in the main text of the work.

α-MoO_3 sample growth and preparation. To fabricate the α-MoO_3 samples, bulk α-MoO_3 crystals (from Alfa Aesar) were mechanically exfoliated using Nitto tape (Nitto Denko Co., SPV 224P). To further thin the material, a second exfoliation was carried out by transferring it from the tape to a transparent polydimethylsiloxane (PDMS) layer. The resulting thin slabs were then inspected under an optical microscope to identify uniform sections with the desired thickness (approximately 155 nm) and large surface areas. These selected thin slabs were subsequently transferred onto a SiO_2(300 nm)/Si substrate using the dry transfer technique[1].

Fabrication of metal antennas. High-resolution electron beam lithography (operated at 100 kV and 100 pA) was carried out with submicron precision on a sample coated with a poly-(methyl methacrylate) (PMMA) resist layer. Using a standard high-resolution developer (a 1:3 mixture of methyl isobutyl ketone (MIBK) and isopropyl alcohol (IPA)), 5 nm of Cr and 30 nm of Au were evaporated, followed by a lift-off process, to define the antennas. To remove any remaining organic material, the sample was treated with a hot acetone bath at 60 °C for 10–15 minutes, gently rinsed with IPA for 1 minute, and dried using nitrogen gas before thermal evaporation. The resulting gold antenna dimensions were 3.3 μm (length) × 286 nm (width) × 40 nm (height).

Scattering-Scanning Near Field Optical Microscopy. Near-field imaging measurements were conducted using a commercial scattering-type Scanning Near Field Optical Microscope (s-SNOM) from Neaspec GmbH, equipped with an Optical Parametric Oscillator (OPO) laser (model PT277-XIR-AOM-B from EKSPLA whose emission ranges from 1.4 to 18 μm thanks to a Difference Frequency Generation (DFG) stage). Metal-coated (Pt/Ir) atomic force microscopy (AFM) tips, operating at a tapping frequency of $\Omega \sim 280$ kHz and with an oscillation amplitude of ~ 150 nm, served as both the source and probe for polaritonic excitations. The gold antennas and AFM tip were illuminated with p-polarized infrared light from the laser. The incident electric field was focused at the extremities of the antenna, causing them to act as two separate point dipoles. The light scattered by the tip was focused by a parabolic mirror into an infrared detector (Kolmar Technologies). To suppress background signals, demodulation of the detected signals $n\Omega$, which can be expressed as the complex-valued functions $\sigma_n = s_n e^{i\phi_n}$, was performed at the 3rd harmonic (n = 3) of the tip's frequency. A pseudo-heterodyne interferometric technique was used to independently measure both the amplitude (s_3) and phase (ϕ_3) signals.

Full-wave numerical simulations. Full-wave numerical simulations were carried out using COMSOL Multiphysics software, based on the finite boundary element method. In the first type of simulations (Figure 1 and Figure 2 c-j of the main text), the model consisted of two semi-infinite media (superstrate and substrate) with an anisotropic thin slab (a hypothetical polaritonic material in Figure 1 and α-MoO_3 in Figure 2 c-j) in between. A vertically oriented electric dipole placed on top of the thin slab acted as a polaritonic launcher. In the second type of simulations (Figure 3f-I of the main text), the structure comprised a semi-infinite superstrate (air), a thin α-MoO_3 slab with a gold antenna on top, and a semi-infinite SiO_2 substrate. The thickness of the thin slab was set to 155 nm, while the gold antenna had dimensions of 3.3 μm in length, 40 nm in height,

and 286 nm in width. These antenna-based simulations involved illuminating the structure with a normally incident plane wave, polarized along the longitudinal axis of the metallic launcher. The permittivity of SiO_2 was taken from ref.[2].

Transfer-Matrix numerical simulations. The Transfer-Matrix method based on ref.[3] was used to obtain the IFCs in Figure 1 of the main text. We have considered the thickness of the anisotropic thin slab to be 200 nm.

Note S2. Calculation of the polariton electric field distribution and the Poynting vector direction.

In this Note, we show numerical calculations of the absolute value of the out-of-plane electric field distributions $|E_z|$ of Figure 1 of the main text. We also show the direction of the Poynting vectors associated with the high-momentum components (see top panels in Figure 1 for clarity). We corroborate the orthogonality of the in-plane Poynting vectors to the corresponding IFCs. The results are summarized in Figure S1.

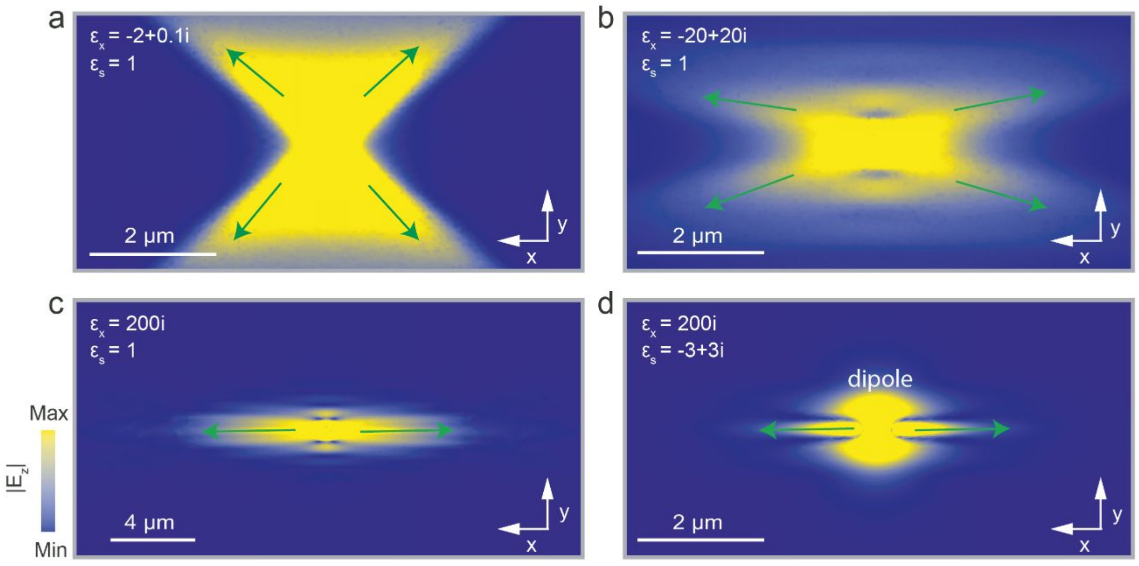

Figure S1. Numerical calculations of the absolute value of the out-of-plane electric field distributions and Poynting vector directions. The green arrows represent the propagation direction of the in-plane Poynting vectors **S** corresponding to the high-momentum components.

Note S3. Quantitative analysis of the Density of Optical States (DOS).

The density of optical states (DOS) excited by a vertically-polarized point-dipole in an anisotropic material is related to the dyadic Green's function component $G_{F,zz}(\boldsymbol{q})$ as[4,5]

$$DOS(\boldsymbol{q}) \propto G_{F,zz}(\boldsymbol{q}), \quad \text{(S1)}$$

where

$$G_{F,zz}(\boldsymbol{q}) \propto q * r_p(\boldsymbol{q}). \text{ (S2)}$$

In Eq. (S2), $r_p(\boldsymbol{q})$ denotes the out-of-plane Fresnel reflection coefficient, which under the high-momentum approximation[6] and for a single anisotropic slab is given by

$$r_p(\boldsymbol{q}) = \frac{(\varepsilon_s - \varepsilon_S)q - \left(\varepsilon_z q_z + \frac{\varepsilon_s q}{\varepsilon_z q_z}\right) tan(q_z k_0 d)}{(\varepsilon_s + \varepsilon_S)q - \left(\varepsilon_z q_z - \frac{\varepsilon_S q}{\varepsilon_z q_z}\right) tan(q_z k_0 d)}, \quad . \text{ (S3)}$$

With ε_s and ε_S the permittivity of the substrate and superstrate, respectively, q_z the out-of-plane momentum component[6], k_0 the free-space wavevector, and d the thickness of the slab. Thus, a quantitative analysis of the DOS can be obtained by studying the Green's Function in Eq. (S2). In particular, we analyze the case exposed in Figure 1d of the main text, which showcases the propagation of in-plane ray polaritons.

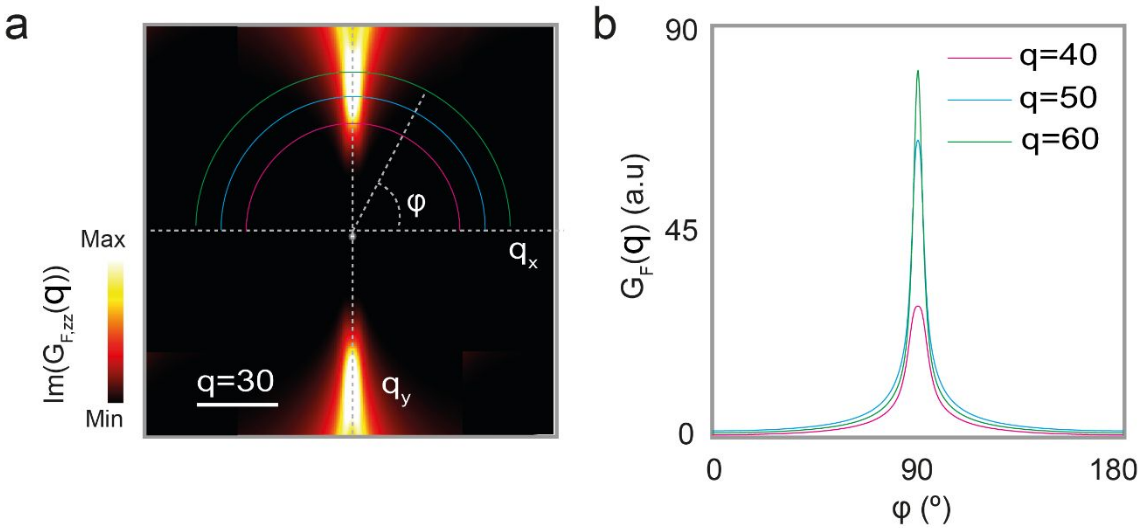

Figure S2. Quantitative analysis of the DOS in Figure 1d. (a) Green's function calculated with Eq. (S2) for the parameters of Figure 1d. The angle φ defines an arbitrary direction of propagation with regards to the x direction. The magenta, blue and green semi-circumferences have 3 different radii of q = 40, q = 50, and q = 60, respectively. (b) Calculation of Eq. (S2) for fixed radii along the semicircular sectors depicted in Figure S2a. Magenta, blue, and green lines correspond to q = 40, q = 50, and q = 60, respectively.

In Figure S2, we demonstrate that the DOS in canalized ray polaritons is highly focused in a narrow angular sector centered along the y direction (φ = 90º).

Also, we have calculated the DOS for different cases corresponding to a transition from a hyperbolic to a canalized IFC (Figure S3a to S3c). As it is difficult to quantitatively

compare the DOS for these different cases, since the permittivity varies strongly both in its real and imaginary components, we have normalized the DOS values. We thus aim on a direct comparison of the DOS angular spreading, observing a clear concentration (Figure S3d) into one specific direction (φ = 90º) in the canalization regime.

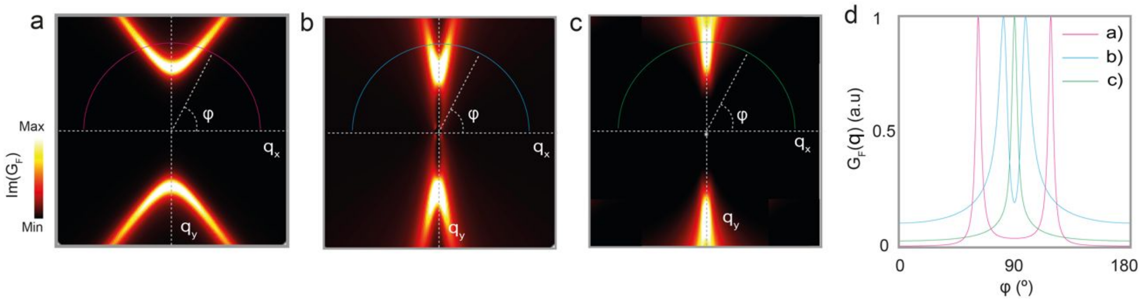

Figure S3. Comparison of the Green's function corresponding to Figure 1a, Figure 1b and Figure 1d. (a) Green's function calculation corresponding to the parameters of Figure 1a. (b) Same as (a) for the parameters of Figure 1b. (c) Same as (a) for the parameters in Figure 1d. (d) Calculation of Eq. (S2) for fixed radii along the semicircular sectors depicted in Figures S2a-S2c. In each case, the calculations have been normalized to the maximum value.

Note S4. Role of the substrate in the canalization of ray PhPs.

As predicted in the main text (Figure 1), the dielectric environment surrounding a thin slab supporting surface polaritons plays a key role in achieving their ray-like canalization. Specifically, we observe that a moderate metallicity of the substrate prevents the appearance of the smallest wavevectors in the polaritonic IFC (top panel in Figure 1c), keeping the high-momentum contributions (see IFC in Figure 1d). This results in the excitation of constant-phase polaritons, i.e., ray polaritons. To further analyze the effect of the substrate on the polaritonic IFC and the corresponding real-space propagation, we show in Figure S4 numerical calculations comparing two very different substrate permittivities ($\varepsilon_s = -3 + 3i$, and $\varepsilon_s = (-3 + 3i)*10^3$).

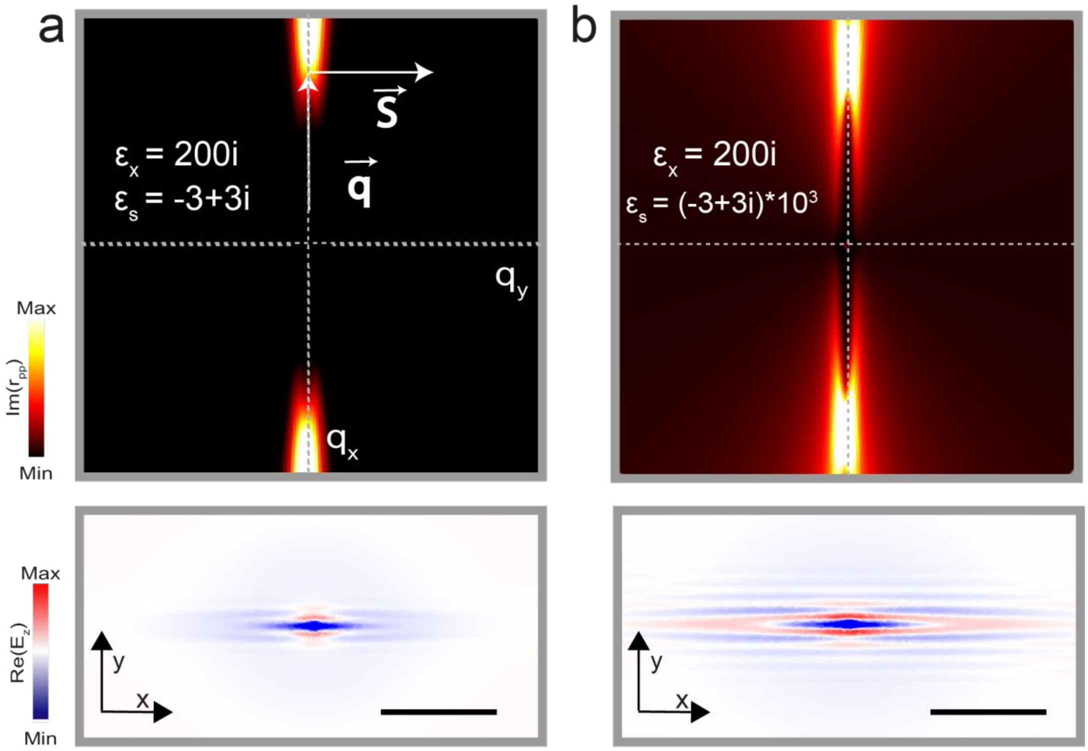

Figure S4. Effect of the substrate permittivity in the propagation of ray-like polaritons. (a) Calculated IFC (top panel) and corresponding real-space electric field distribution, Re(E_z), (bottom panel) of surface polaritons in a single in-plane anisotropic slab with permittivity ε_x = 200i, $\varepsilon_y = -1 + 0.1i$, and $\varepsilon_z = 5 + 0.1i$ surrounded by air ($\varepsilon_s = 1$) and a substrate with $\varepsilon_s = -3 + 3i$. The thickness of the slab is set to d = 200 nm. The scale bar in the bottom panel is 2 µm. (b) Same as (a) for $\varepsilon_s = -3000 + 3000i$.

Figure S4a reproduces the results shown in Figure 1d of the main text. In stark contrast, Figure S4b shows that a much larger negative value of the substrate permittivity ε_s = -3000 + 3000i prevents the ray-like propagation of the polaritons. Instead, we observe an elliptical propagation. Thus, a compromise on searching moderate values of the substrate permittivity modulus becomes crucial to obtain well-defined in-plane ray propagation (as such, metallic substrates with a high modulus of the permittivity, e.g. Au, are not suitable candidates).

Figure S5 analyzes the effect of the substrate in the case of a thin polar slab (α-MoO_3) supporting PhPs. The images, calculated at the α-MoO_3 RB2 TO phonon frequency (ω_3 = 821 cm^{-1}), consider SiO_2 (Figure S5a) and gold (Figure S5b) as substrates, i.e., two media with very different permittivities at the frequency considered. For SiO_2 (moderate values of the permittivity), the results show the propagation of canalized ray PhPs, as shown in the main text (Figure 2e). In contrast, in the case of using gold (large values of the permittivity), the excited PhPs show a clear elliptical propagation. These results corroborate the important role that the permittivity of the substrate plays in the excitation of ray polaritons exhibiting canalization.

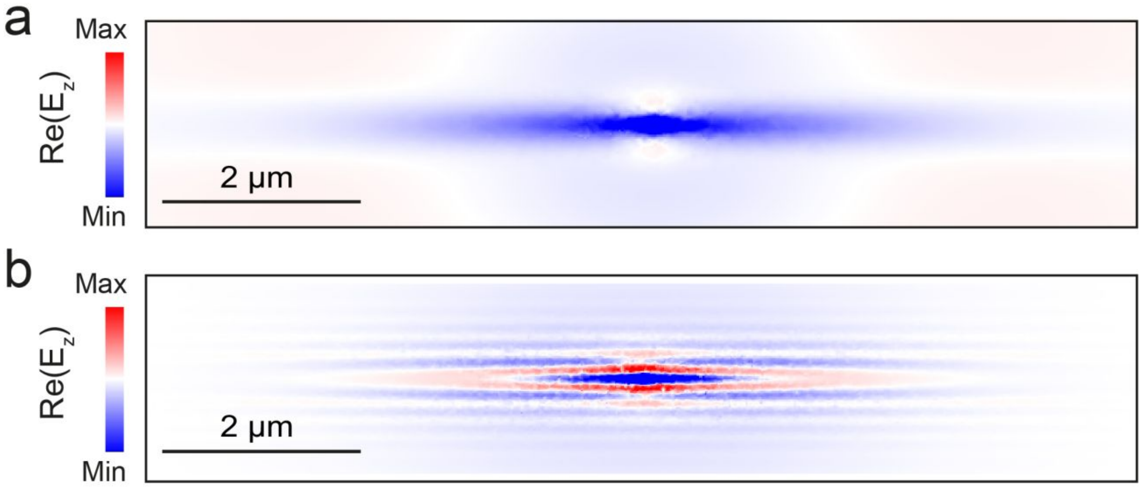

Figure S5. Comparison of the PhPs propagation in α-MoO_3 at ω = 821 cm^{-1} for two substrates (SiO_2 and Au) with very different permittivity values. (a) Real-space electric field distributions, Re(E_z), when the substrate is SiO_2. The thickness of the slab is set to d =155 nm, (b) Same as (a) for Au.

Note S5. Discussion of the structural anisotropy of α-MoO_3.

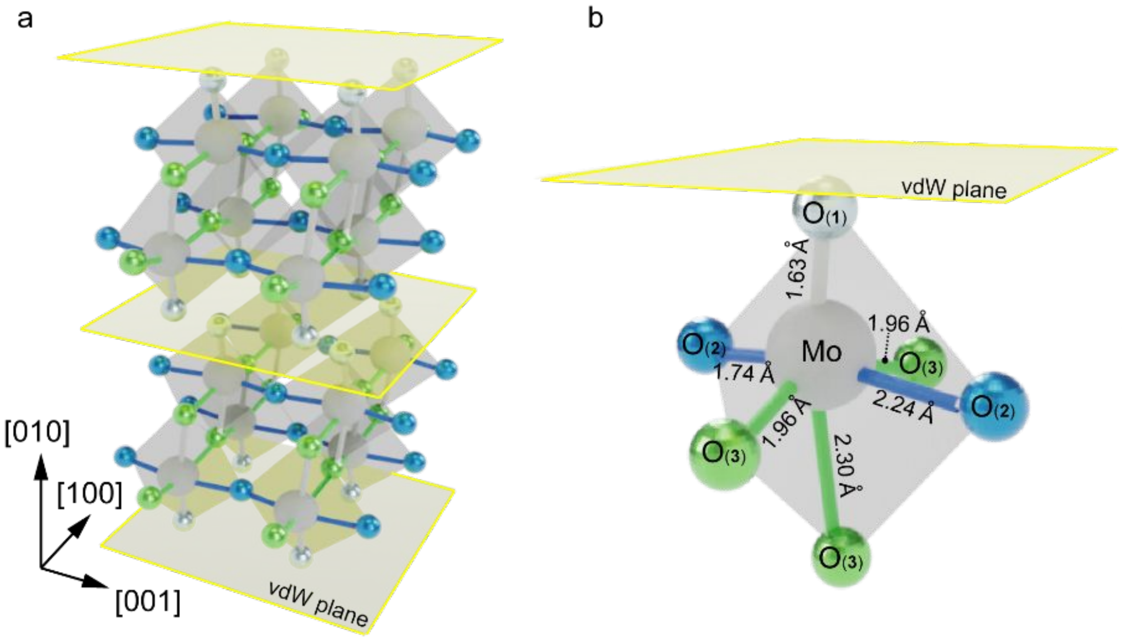

Figure S6. Structural anisotropy in α-MoO_3. (a) Crystal structure of α-MoO_3. Dull grey spheres represent molybdenum (Mo) atoms —the largest spheres in the figure—, and metallic blue, green, and grey spheres represent oxygen (O) atoms distinctly linked to the Mo atoms. The vdW plane indicates the spatial region where the α-MoO_3 monolayers are weakly bonded by vdW forces. (b) The three symmetry-distinct oxygen atoms[5,6] in each MoO_6 octahedron are labeled as $O_{(1)}$ (metallic green spheres), $O_{(2)}$ (metallic blue spheres), and $O_{(3)}$ (metallic grey spheres).

The α-MoO_3 is a layered compound whose orthorhombic crystal structure comprises double layers of distorted MoO_6 octahedra[7], as depicted in Figure S6. These double layers stack along the [010] crystal direction and are weakly bonded across a region known as the van der Waals plane[8]. Six oxygen (O) atoms coordinate each molybdenum (Mo) atom, but these oxygen atoms are not equivalent. They are classified into three distinct types based on their bonding environment and spatial arrangement around the Mo atom as follows. (i) **Terminal oxygen atom ($O_{(1)}$)**, located at the apex of the octahedra within the vdW plane, forming a double bond with a single Mo atom, denoted as Mo=O, with a strong and short bond with a length of 1.63 Å[9]. (ii) **Corner-sharing oxygen atoms ($O_{(2)}$)** bridge between two Mo atoms by sharing corners of the octahedra along the [001] crystallographic direction. These O atoms facilitate the formation of zig-zag chains within the layers, and their Mo–O bond lengths range from 1.74 Å to 2.24 Å[9]. (iii) **Edge-sharing oxygen atoms ($O_{(3)}$)** bridge between two Mo atoms by sharing edges of the octahedra along the [010] direction and the Mo–O bond lengths are the longest, ranging from 1.96 Å to 2.30 Å[9].

Note S6. Investigation of the influence of slab thickness on the propagation of canalized ray PhPs in α-MoO_3/SiO_2.

To understand whether the thickness of the α-MoO_3 slab plays an important role in the propagation of canalized ray PhPs, we have performed numerical simulations in α-MoO_3/SiO_2 at $\omega = 821\ cm^{-1}$ for different thicknesses. The results are summarized in Figure S7.

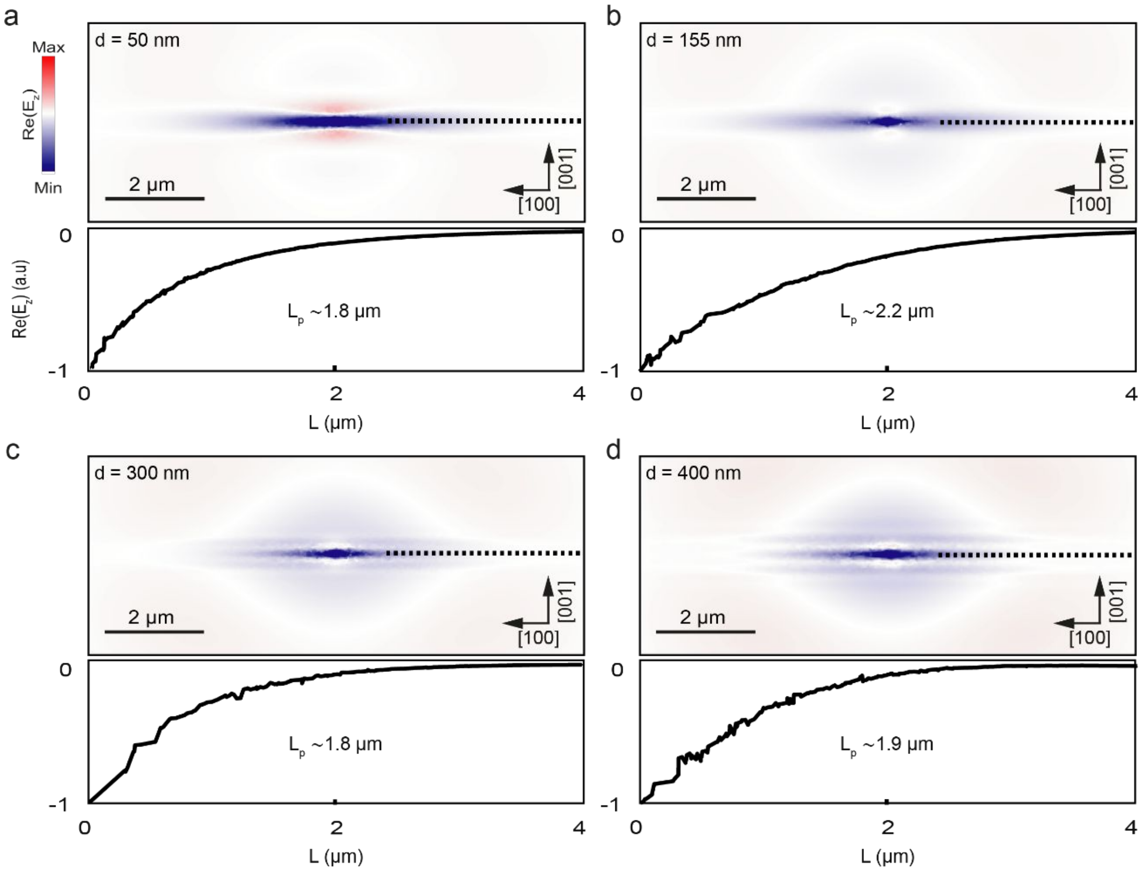

Figure S7. Influence of α-MoO_3 slab thickness in the propagation of canalized ray PhPs in α-MoO_3/SiO_2. Propagation of canalized ray PhPs in α-MoO_3/SiO_2 at ω=821 cm^{-1} while varying the α-MoO_3 slab thickness, d, as (a) d = 50 nm, (b) d=155 nm, (c) d = 300 and (d) d = 400nm. In all panels we show the real space electric field distribution Re(E_z) in the XY plane (top) and the electric field value Re(E_z) along the profiles depicted by dashed black lines (top). An estimation of the propagation length L_p is given in the bottom panels.

We observe no significant differences in the propagation of canalized ray polaritons along the [100] direction for the different thicknesses. Importantly, the propagation length, taken as the distance at which the electric field modulus decays a factor 1/e, shows no important variations. We attribute the discrepancies in the values to the meshing of the numerical calculations. Remarkably, as the thickness increases, we observe polaritonic fringes propagating along the [001] in-plane direction. These oscillations are the out-of-plane hyperbolic rays in the YZ direction, which become easier to resolve as the thickness is increased (i.e, the peak-to-peak distance increases with the slab thickness).

Note S7. Constant propagating phase of canalized ray polaritons.

Figure S8 shows the simulated real part of the electric field distribution (top), its phase (middle) and a phase profile (bottom, corresponding to the white dashed line in the middle panel, which is taken 1 μm away from the point-dipole source to properly capture the polaritonic contribution to the electromagnetic field).

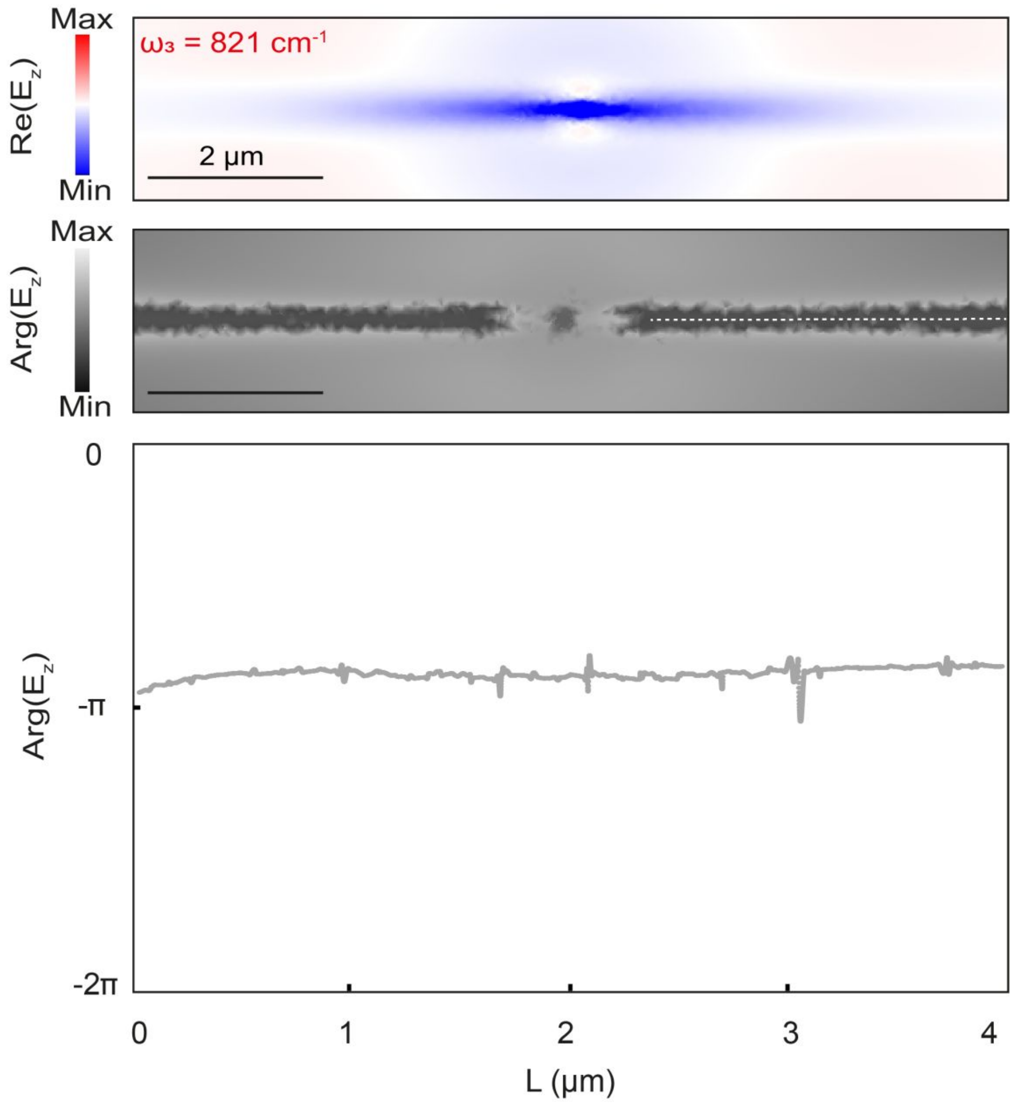

Figure S8. Electric field phase of canalized ray polaritons. Real part of the electric field Re(E_z) (top), phase arg(E_z) (middle), and phase along the profile depicted by a white dashed line in the middle panel (bottom).

We can clearly observe that the phase takes a nearly constant value close to -π, ensuring the constant phase behavior of canalized ray polaritons (we attribute the observed sharp deviations to irregularities in the meshing used to obtain the numerical simulation).

Note S8. Investigation of the influence of material losses along the α-MoO_3 [100] in-plane direction on the propagation of canalized ray PhPs in α-MoO_3/SiO_2.

To investigate the role of material losses in the PhP propagation within α-MoO_3 at ω_3 = 821 cm^{-1}, where the permittivity along its [100] in-plane direction is $\varepsilon_{[100]} = 39.79 + 272.56i$[10], we calculate the real-space propagation of in-plane PhPs launched by a point dipole in the α-MoO_3/SiO_2 system. We varied the imaginary part of ε_x from $Im(\varepsilon_{[100]}) = 0$ to $Im(\varepsilon_{[100]}) = 272.56$ while keeping the real part fixed at $Re(\varepsilon_{[100]}) = 39.79$. This approach allowed us to systematically analyze how increasing material losses along the [100] direction affect PhP propagation at this specific frequency.

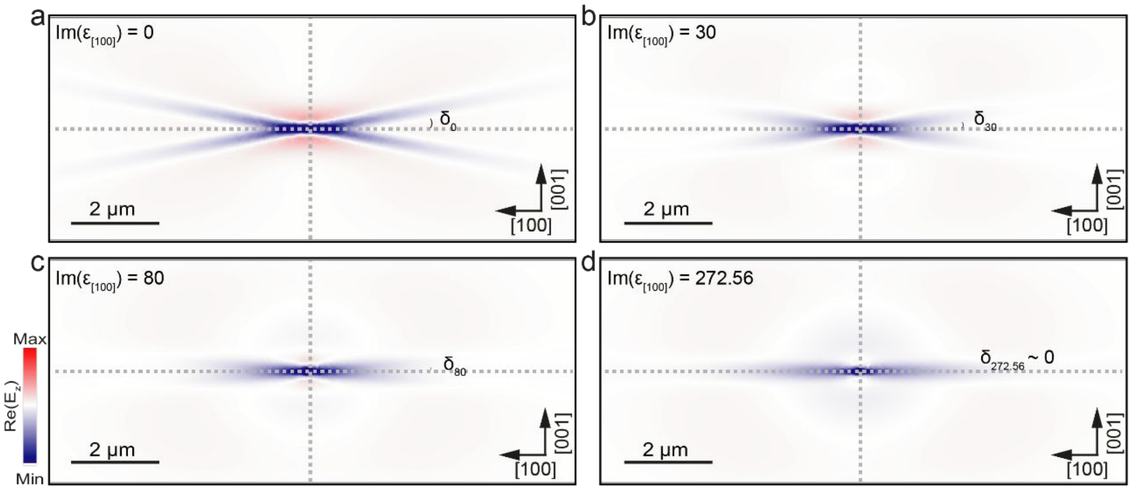

Figure S9. Influence of material losses on the propagation of canalized ray PhPs in α-MoO_3/SiO_2. Propagation of canalized ray PhPs in α-MoO_3/SiO_2 at 821 cm^{-1} while varying the imaginary part of the α-MoO_3 permittivity along the [100] in-plane crystal direction ($\varepsilon_{[100]}$) as (a) $Im(\varepsilon_{[100]}) = 0$ (loss-less case), (b) $Im(\varepsilon_{[100]}) = 30$, (c) $Im(\varepsilon_{[100]}) = 80$ and (d) $Im(\varepsilon_{[100]}) = 272.56$ (experimental real value). As $Im(\varepsilon_{[100]})$ increases, the ray PhP propagation collimates towards the [100] in-plane crystal direction.

The resulting simulated images were compiled into a continuous sequence, presented in the following video [link to the video]. Four illustrative cases are shown in Figure S9. At the beginning of the video, representing the loss-less case ($Im(\varepsilon_{[100]}) = 0$, see Figure S9a), PhPs propagate as two polaritonic rays along a direction that is oblique to the [100], forming a non-zero angle, δ, between both directions. This type of ray PhP propagation is similar to that observed in ref.[11]. Interestingly, as $Im(\varepsilon_{[100]})$ increases (Figure S9b,c), the polaritonic rays gradually collimate towards the [100] direction, thus decreasing δ. Finally, when $Im(\varepsilon_{[100]}) = 272.56$ (Figure S9d), PhP propagation becomes fully collimated along the [100] direction and δ = 0, resulting in the canalization of ray PhPs along this precise direction. Overall, this simulation study confirms the crucial role that material losses play in observing canalized ray polaritons in a single thin slab of α-MoO_3 on SiO_2.

Supporting Information references

(1) Castellanos-Gomez, A.; et al. Deterministic transfer of two-dimensional materials by all-dry viscoelastic stamping. *2D Mater.* **2014**, *1*, 011002.

(2) Aguilar-Merino, P.; Álvarez-Pérez, G.; Taboada-Gutiérrez, J.; et al. Extracting the Infrared Permittivity of SiO_2 Substrates Locally by Near-Field Imaging of Phonon Polaritons in a van der Waals Crystal. *Nanomaterials* **2021**, *11*, 120.

(3) Passler, N.C. and Paarmann, A. Generalized 4 × 4 matrix formalism for light propagation in anisotropic stratified media: study of surface phonon polaritons in polar dielectric heterostructures. *J. Opt. Soc. Am. B* **2017**, *34*, 2128-2139.

(4) Duan, J.; et al. Enabling propagation of anisotropic polaritons along forbidden directions via a topological transition. *Sci. Adv.* **2021**, *7*, eabf2690.

(5) Martín-Sánchez, J.; et al. Focusing of in-plane hyperbolic polaritons in van der Waals crystals with tailored infrared nanoantennas. *Sci. Adv.* **2021**, *7*, eabj0127.

(6) Álvarez-Pérez, G.; et al. Analytical approximations for the dispersion of electromagnetic modes in slabs of biaxial crystals. *Phys. Rev. B* **2019**, 100, 235408.

(7) Ma, W.; Alonso-González, P.; Li, S.; et al. In-plane anisotropic and ultra-low-loss polaritons in a natural van der Waals crystal. *Nature* **2018**, *562*, 557–562.

(8) Ding, H.; et al. Structural and vibrational properties of α-MoO_3 from van der Waals corrected density functional theory calculations. *Phys. Rev. B.* **2012**, *85*, 012104

(9) Negishi, H.; et al. Anisotropic thermal expansion of layered MoO_3 crystals. *Phys. Rev. B.* **2004**, *69*, 109902.

(10) Álvarez-Pérez, G.; et al. Infrared Permittivity of the Biaxial van der Waals Semiconductor α-MoO_3 from Near- and Far-Field Correlative Studies. *Adv. Mater.* **2020**, *32*, 1908176.

(11) Álvarez-Cuervo, J.; Obst, M.; Dixit, S.; et al. Unidirectional ray polaritons in twisted asymmetric stacks. *Nat Commun* **2024**, *15*, 9042.
